# Attitudes towards diversity, equity, and inclusion across the CTSA Programs: Strong but not uniform support and commitment

**DOI:** 10.1017/cts.2022.525

**Published:** 2023-02-07

**Authors:** Jeffrey Duong, Scott McIntosh, Jacqueline Attia, J. Lloyd Michener, Linda B. Cottler, Sergio A. Aguilar-Gaxiola

**Affiliations:** 1 Center for Reducing Health Disparities, University of California – Davis School of Medicine, Sacramento, CA, USA; 2 Center for Leading Innovation and Collaboration, Department of Public Health Sciences, University of Rochester Medical Center, Rochester, NY, USA; 3 Department of Family Medicine & Community Health, Duke School of Medicine, Durham, NC, USA; 4 Department of Epidemiology, Colleges of Public Health and Health Professions and Medicine, University of Florida, Gainesville, FL, USA; 5 Clinical and Translational Science Center, Department of Internal Medicine, School of Medicine, Sacramento, CA, USA

**Keywords:** Health equity, DEI, translational research, community engagement, workforce training

## Abstract

**Background:**

This study describes attitudes towards diversity, equity, and inclusion (DEI) among members of the Clinical and Translational Science Awards (CTSA) Program. It also explores associations between program members’ roles and their perceived importance of and commitment to improving DEI and assesses the link between perceived importance of and commitment to improving DEI. Lastly, it ascertains barriers and priorities concerning health equity research, workforce development, CTSA consortium leadership, and clinical trials participation among respondents.

**Methods:**

A survey was administered to registrants of the virtual CTSA Program 2020 Fall Meeting. Respondents reported their roles, perceived importance of and commitment to improving DEI. Bivariate cross-tabulations and structural equation modeling examined associations between respondents’ roles, perceived importance of DEI, and commitment to improving DEI. Grounded theory was used to code and analyze open-ended questions.

**Results:**

Among 796 registrants, 231 individuals completed the survey. DEI was “extremely important” among 72.7 percent of respondents and lowest among UL1 PIs (66.7%). Being “extremely committed” to improving DEI was reported by 56.3 percent of respondents and lowest among “other staff” (49.6%). Perceived importance of DEI was positively associated with commitment to improve DEI. *Institutional and CTSA Commitment, Support, and Prioritization of DEI* represented a key theme for improving DEI among respondents.

**Conclusion:**

Clinical and translational science organizations must take bold steps to transform individual perceptions of DEI into commitment and commitment into action. Institutions must set visionary objectives spanning leadership, training, research, and clinical trials research to meet the promise and benefits of a diverse NIH-supported workforce.

## Introduction

In 2012, the National Institutes of Health (NIH) established the National Center for Advancing Translational Sciences (NCATS) [1]. Through its Clinical and Translational Science Awards (CTSA) Program, NCATS supports a national network of approximately 60 medical research institutions (i.e., hubs) that work together to foster innovation in training, research, and processes, with the goal of speeding the translation of research discovery and delivering more treatments to more patients more quickly. Over the last decade, the CTSA Program has continued to evolve with new projects and initiatives, including an increased focus on improving diversity, equity, and inclusion (DEI) across research institutions that has been long overdue. To drive further change, in 2014, the NIH funded the National Research Mentoring Network (NRMN) to address the lack of full participation of underrepresented minorities (URMs) and other underrepresented groups across all Biomedical, Behavioral, Clinical and Social Science Research Careers. In 2019, NIH officially declared significant interest in supporting a diverse workforce in the research enterprise [2].

It is widely accepted that diversity in science fosters innovation, enhances global competitiveness, and improves the quality of research and research outcomes [2]. Accordingly, for the 2020 Fall Virtual CTSA Program Meeting, *Diversity, Equity, and Inclusion – Approaches and Solutions in Translational Science* was selected as its main theme [3]. The agenda for the meeting included planned discussions on the importance of DEI in clinical and translational science, emphasizing the need to identify, uncover, and dismantle sources of systemic racism and bias that would undermine DEI in the field. Currently, little is known about the attitudes toward DEI among individual researchers and staff involved in the field of clinical and translational science. Prior to the meeting, the planning committee created and disseminated a survey that would be used as a registrant baseline about the importance of and commitment to DEI and to inform breakout sessions and follow-up meetings that would ultimately produce recommendations for sustainable change [4].

We report on findings from the pre-meeting survey for the 2020 Fall Virtual CTSA Program Meeting [5]. Specifically, we investigate the extent to which CTSA Program members perceived DEI to be important, as well as the extent to which members were committed to improving DEI through fundamental changes in the way the consortium operates. We also examined whether CTSA members’ attitudes toward DEI differed based on their roles in the program. Finally, we assessed the association between perceived importance of DEI and commitment to improving DEI through fundamental changes among the CTSA Program members. These findings could have implications regarding the development of programs and initiatives that advance DEI and diversify the translational research workforce within the CTSA program consortium.

## Methods

### Procedure

A pre-meeting survey was developed and pre-tested prior to implementation. The survey contained a combination of single- and multiple-response items, Likert scales, and short open-text response questions. A penultimate survey draft was circulated to the research team, who beta-tested the draft and provided critical feedback that informed a revised final survey. Throughout the survey development process, the Working Group sought to balance obtaining a rich data set against overburdening survey respondents. The 2020 Fall CTSA Program Meeting was held in November 2020. One week prior, a web link to the final voluntary survey (Table 1) was emailed to all meeting registrants across the more than 60 active CTSA hubs. Two reminders were sent to encourage completion. A total of 231 individuals completed the survey out of 796 registrants, resulting in a 29.0 percent response rate.

**Table 1.** Clinical and Translational Science Awards (CTSA) Program 2020 Fall Pre-meeting survey items

**Survey item**	**Response options**
Which of the following best describes you? (Check All That Apply)	UL1 PI UL1 Executive Director/Administrator KL2 Director TL1 Director CTSA Program Hub Steering Committee Member NCATS Program Officer or Representative Other
How important is diversity, equity, and inclusion (DEI) to you?	Not at all important (1) → Extremely Important (5)
How committed are you to improving DEI through making fundamental changes in the way the consortium operates?	Not at all committed (1) → Extremely Committed (5)
Please list the **Top Barrier** that has significantly limited your sphere of influence for the following categories:	Workforce Development CTSA Consortium Leadership Disparities/Health Equity Research Clinical Trials Participation
Please list your **Top Priority/Suggestion** to the field for overcoming the barriers that you listed above with regard to the following:	Workforce Development CTSA Consortium Leadership Disparities/Health Equity Research Clinical Trials Participation

UL1 PI = UL1 Principal Investigator; KL2 = Institutional Career Development Core; TLI = Institutional Training Core; NCATS = National Center for Advancing Translational Sciences.

*Note.* A Clinical and Translational Science Award (CTSA) Program hub is defined as an institution in receipt of an UL1 award with a linked KL2 award and an optional TL1 award. For more information, please see: https://ncats.nih.gov/ctsa/about/hubs.

### Survey

The survey addressed respondents’ perceptions toward DEI in the context of the CTSA Program. Table 1 outlines the items from the CTSA Program 2020 Fall Pre-Meeting Survey. Respondents were asked which roles best described them as well as their attitudes toward DEI (e.g., “How **important** is DEI to you?” and “How **committed** are you to improving DEI through fundamental changes in the way the consortium operates?”). The survey also included open-ended items for respondents to list **barriers** and **priorities** regarding the domains of workforce development, CTSA consortium leadership, disparities/health equity research, and clinical trials participation. The survey was reviewed by the University of Rochester Institutional Review Board and determined to be exempt from the requirements of the Code of Federal Regulations.

### Data Analysis

**Fig. 1.**
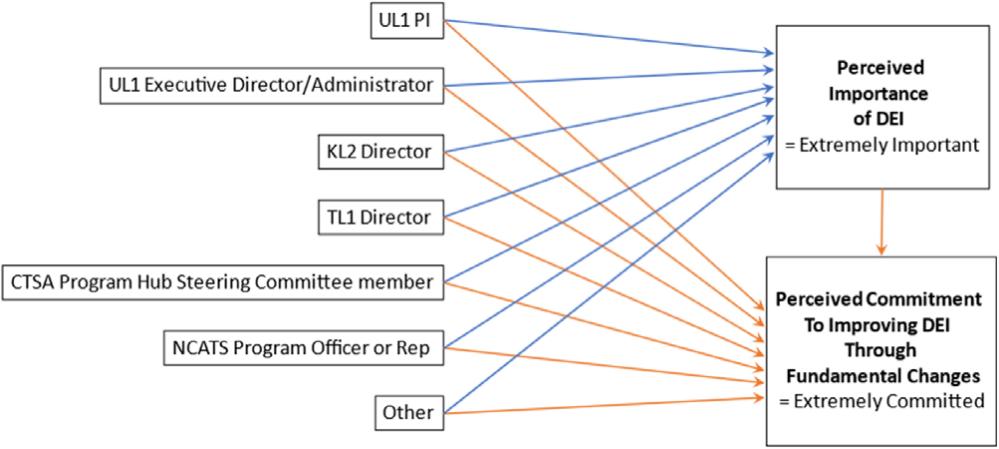
Generalized structural equation model. UL1 PI = UL1 Principal Investigator; KL2 = Institutional Career Development Core; TLI = Institutional Training Core; CTSA = Clinical and Translational Science Award; DEI = diversity, equity, and inclusion; NCATS = National Center for Advancing Translational Sciences.

***Quantitative analysis*.** Data from respondents were collected and managed using REDCap [6] electronic data capture tools hosted at the University of Rochester Center for Leading Innovation and Collaboration (CLIC). Descriptive analyses were performed on aggregate de-identified data from all respondents. The quantitative data were analyzed using Stata version 16^7^. Univariate tabulations were used to obtain sample characteristics. Bivariate cross-tabulations were used to describe respondents’ perceived importance of DEI across their various roles. Cross-tabulations were also used to describe respondents’ perceived commitment to improving DEI across their various roles. We also constructed a path model **(**Fig. 1 **)** to examine associations between respondents’ roles within the CTSA consortium and their perceived importance of DEI and perceived commitment to improving DEI. With regard to perceived importance of DEI and perceived commitment to improving DEI, the path model compared the odds of respondents identifying that DEI was “extremely” important to them or that they were “extremely” committed to DEI. The rationale behind this is that future DEI efforts should strive toward these goals among CTSA Program members. Secondly, because nearly all respondents selected the “extremely” or “very” important/committed answer choices, it is important to gain an understanding on where there may be differences in which respondents were more likely to select the “extremely important” or “extremely committed” answer choices over others. Individuals who identified themselves as having a particular role were compared to all others as the reference group (e.g., UL1 PIs vs. Non-UL1 PIs), because respondents had the option to identify as multiple roles and therefore the role categories were not mutually exclusive. Finally, we assessed the association between respondents’ perceived importance of DEI and commitment to improving DEI. A path analysis was conducted in a generalized structural equation modeling (SEM) [8] framework to allow multiple associations to be tested simultaneously; a maximum likelihood estimator computed standard errors that were robust to non-normality of observations.

***Qualitative analysis*.** Survey data were exported from REDCap to an Excel spreadsheet. Several questions invited text responses for qualitative analysis. Personal and institutional identifiers were redacted from text responses prior to analysis. After establishing a general framework for examining qualitative data (open coding of text related to initial domains of interest), an axial coding strategy based on the grounded theory approach[9] led to specific categories, following procedures that the authors have used previously [10–12]. Two experienced coders independently assigned initial codes to each text response; a third individual served as an additional decision maker as needed. After coding, review, and regular peer debriefing, themes that emerged were organized within broader domains when applicable and compared among and between domain areas. A simple proportion agreement method and thematic constant comparative (TCC) approach were used for inter-rater consistency during theme development [13]. Per established procedures for qualitative data analysis [10,11], inter-rater reliability was assessed using the simple proportion agreement method rather than a more complex statistic (e.g., Kappa coefficient). This is due to the large number of thematic codes, the possibility for multiple codes within text segments, and the exploratory nature of this study [13].

## Results

### Participants

Of 796 meeting registrants, 231 respondents from 54 of 60 hubs completed the pre-meeting survey. A little over 15 percent of respondents identified as UL1 Executive Directors or Administrators, followed by 13 percent identifying as UL1 Principal Investigators. The majority of respondents identified their role as “Other” (50.6%), which included non-senior-level administrators, staff, and program directors.

Nearly three out of four respondents (72.7%) indicated that DEI was “extremely important” to them. Furthermore, over half (56.3%) of the respondents indicated that they were “extremely committed” to improving DEI through making fundamental changes in the way the CTSA consortium operated. Overall, 93.5 percent of respondents indicated that DEI was either very or extremely important to them. Furthermore, 86.2 percent of respondents indicated that they were very or extremely committed to improving DEI.

### Perceived Importance of DEI and Commitment to Improving DEI

Tables 2 and 3 present the attitudes toward DEI among leaders of the CTSA hubs. The proportion of respondents indicating that DEI was “extremely important” to them was highest among NCATS Program Officers or Representatives (100.0%), followed by TL1 Directors (86.7%) and CTSA Program Hub Steering Committee members (83.3%) (Table 2). It was lowest among UL1 PIs (66.7%), UL1 Executive Directors/Administrators (71.4%), as well as respondents who identified their role as “other” (70.9%; e.g., non-senior-level administrators, staff, and other program directors). Indeed, the UL1 Executive Director/Administrator and other staff groups were the only to have respondents who reported that DEI was only “somewhat important” to them. With regard to the proportion of respondents indicating that they were “extremely committed” to improving DEI through making fundamental changes (Table 3), this was also lowest among UL1 PIs, UL1 Executive Directors/Administrators, as well as other staff (ranging from 49.6 to 56.7%). Moreover, UL1 PIs and other staff were the only groups to report that they were only somewhat committed to improving DEI.

**Table 2.** Perceived importance of diversity, equity, and inclusion among respondents by role (*N* = 231)

	*How important is diversity, equity, and inclusion (DEI) to you?*					
	Not at all important *n* (%)	Somewhat important *n* (%)	Important *n* (%)	Very important *n* (%)	Extremely important *n* (%)	Total *n* (%)
UL1 PI	0 (0.0)	0 (0.0)	2 (6.7)	8 (26.7)	20 (66.7)	30 (100.0)
UL1 Executive Director or Administrator	0 (0.0)	1 (2.9)	2 (5.7)	7 (20.0)	25 (71.4)	35 (100.0)
KL2 Director	0 (0.0)	0 (0.0)	0 (0.0)	5 (22.7)	17 (77.3)	22 (100.0)
TL1 Director	0 (0.0)	0 (0.0)	0 (0.0)	2 (13.3)	13 (86.7)	15 (100.0)
CTSA Program Hub Steering Committee Member	0 (0.0)	0 (0.0)	0 (0.0)	3 (16.7)	15 (83.3)	18 (100.0)
NCATS Program Officer or Representative	0 (0.0)	0 (0.0)	0 (0.0)	0 (0.0)	7 (100.0)	7 (100.0)
Other Staff	0 (0.0)	2 (1.7)	8 (6.8)	24 (20.5)	83 (70.9)	117 (100.0)
Total	0 (0.0)	3 (1.3)	12 (5.2)	48 (20.8)	168 (72.7)	231 (100.0)

UL1 = NIH grant mechanism; UL1 PI = UL1 Principal Investigator; KL2 = Institutional Career Development Core; TLI = Institutional Training Core; CTSA = Clinical and Translational Science Award; NCATS = National Center for Advancing Translational Sciences.

**Table 3.** Commitment to diversity, equity, and inclusion (DEI) among respondents by role (N = 231)

	*How committed are you to improving DEI through making fundamental changes?*					
	Not at all committed *n* (%)	Somewhat committed *n* (%)	Committed *n* (%)	Very committed *n* (%)	Extremely committed *n* (%)	Total *n* (%)
UL1 PI	0 (0.0)	1 (3.3)	2 (6.7)	10 (33.3)	17 (56.7)	30 (100.0)
UL1 Executive Director or Administrator	0 (0.0)	0 (0.0)	6 (17.1)	9 (25.7)	20 (57.1)	35 (100.0)
KL2 Director	0 (0.0)	0 (0.0)	1 (4.5)	5 (22.7)	16 (72.7)	22 (100.0)
TL1 Director	0 (0.0)	0 (0.0)	0 (0.0)	6 (40.0)	9 (60.0)	15 (100.0)
CTSA Program Hub Steering Committee Member	0 (0.0)	0 (0.0)	1 (5.6)	4 (22.2)	13 (72.2)	18 (100.0)
NCATS Program Officer or Representative	0 (0.0)	0 (0.0)	0 (0.0)	2 (28.6)	5 (71.4)	7 (100.0)
Other Staff	0 (0.0)	1 (0.9)	19 (16.2)	39 (33.3)	58 (49.6)	117 (100.0)
Total	0 (0.0)	2 (0.9)	30 (13.0)	69 (29.9)	130 (56.3)	231 (100.0)

UL1 PI = UL1 Principal Investigator; KL2 = Institutional Career Development Core; TLI = Institutional Training Core; CTSA = Clinical and Translational Science Award; NCATS = National Center for Advancing Translational Sciences.

Table 4 shows the respondents’ CTSA role and their attitude and commitment to DEI. Among 30 respondents who identified as a UL1 PI, 20 individuals (66.7%) reported that DEI was “extremely important.” Across all roles, over two-thirds of respondents indicated that DEI was extremely important. All respondents who identified as NCATs Program Officers or Representatives reported that DEI was extremely important to them. There were no significant associations between respondents’ roles and the perceived importance of DEI.

**Table 4.** Associations between CTSA role and attitude towards importance and commitment to diversity, equity, and inclusion

	Diversity, equity, and inclusion (DEI) is extremely important		Extremely committed to improving DEI through fundamental changes	
	*n* (%)	Odds ratio (95% CI)	*n* (%)	Odds ratio (95% CI)
**Role**				
UL1 PI (vs. Non-UL1 PIs)	20/30 (66.7)	2.16 (0.23–20.51)	17/30 (56.7)	0.45 (0.09–2.24)
UL1 Executive Director or Administrator (vs. Non-UL1 Executive Directors)	25/35 (71.4)	2.49 (0.31–20.16)	20/35 (57.1)	0.42 (0.10–1.80)
KL2 Director (vs. Non-KL2 Directors)	17/22 (77.3)	3.09 (0.24–40.18)	16/22 (72.7)	1.48 (0.30–7.28)
TL1 Director (vs. Non-TL1 Directors)	13/15 (86.7)	5.75 (0.29–114.48)	9/15 (60.0)	0.23 (0.05–1.03)
CTSA Program Hub Steering Committee Member (vs. Non-CTSA Program Hub Steering Committee Members)	15/18 (83.3)	4.18 (0.63–27.79)	13/18 (72.2)	0.90 (0.18–4.58)
NCATS Program Officer or Representative (vs. Non-NCATS Program Officers or Representatives)	7/7 (100.0)	Not Calculable	5/7 (71.4)	0.23 (0.03–1.74)
Other Staff (vs. Staff not identifying as “Other”)	83/117 (70.9)	2.62 (0.31–22.06)	58/117 (49.6)	0.21 (0.05–0.87)
**How Important is Diversity, Equity, and Inclusion?**				
Extremely Important (vs. Not Extremely Important)	--	--	128/168 (76.2)	131.41 (23.91–722.23)

UL1 PI = UL1 Principal Investigator; KL2 = Institutional Career Development Core; TLI = Institutional Training Core; CTSA = Clinical and Translational Science Award; NCATS = National Center for Advancing Translational Sciences.

*Notes.* CI = Confidence Interval. CFI/TLI = 1.00; RMSEA < 0.001; SRMR < 0.001.

For those respondents who identified as a UL1 PI, 17 individuals (56.7%) reported that they were “extremely committed” to improving DEI through making fundamental changes in the way the CTSA consortium operates. Across all roles, approximately half or more of the respondents indicated that they were extremely committed to improving DEI. In our path model, however, respondents who identified their role as “other” had a significantly lower odds of being extremely committed to improving DEI through fundamental changes compared to those who did not indicate “other” (odds ratio [OR] = 0.21; 95% confidence interval [CI] = 0.05, 0.87).

Among the 168 respondents who reported that DEI was extremely important to them, 128 individuals (76.2%) were extremely committed to improving DEI through fundamental changes. Those who reported that DEI was extremely important to them had a significantly greater odds of being extremely committed to improving DEI through fundamental changes compared to those who did not report that DEI was extremely important (OR = 126.48; 95% CI = 23.00, 695.61).

### Barriers and Priorities for Disparities and Health Equity Research, Workforce Development, CTSA Consortium Leadership, and Clinical Trials Participation

Several themes emerged concerning the barriers as well as priorities or suggestions for health disparities research, workforce development, consortium leadership, and clinical trials participation in the CTSA Program (Table 5).

**Table 5.** Barriers and priorities for Disparities and Health Equity Research, workforce development, CTSA consortium leadership, and clinical trials participation

Areas of interest	Representative comments
***Disparities and Health Equity Research***	
**-- Barriers (*n* = 138)**	
*Funding and Resources (n = 42)*	“Limited money to support the high level of interest and competence among faculty and community partners to conduct partnered research”
*Institutional and CTSA Commitment, Support, and Prioritization of DEI (n = 26)*	“Difficult to shift work towards this focus as hub has not made explicit how we will implement DEI in work” “Lack of time/priority from top down – usually existing faculty working extra to meet a need”
*DEI Faculty and Staff Outreach, Recruitment, Retention, and Advancement (n = 24)*	“Lack of researchers engaged in this field” “Limited by number of experienced investigators who can provide mentorship to others” “Current lack of diversity in faculty”
*Awareness, Understanding, and Knowledge (n = 15)*	“Increasing access to diverse populations” “Lack of awareness of research in this area” “Getting others to understand it is a priority”
*Community Engagement or Lack of Trust in Community (n = 8)*	“Low levels of trust in the institution and trust in research from diverse communities”
*Other (n = 23)*	“APT requirements and need to publish early and often” “Lack of fit within current program” “Creativity” “Structural problems in US and state policy”
**-- Priorities or Suggestions (*n* = 135)**	
*Funding and Resources (n = 41)*	“Explicit funding mechanism outside of NIMHD”
*Institutional and CTSA Commitment, Support, and Prioritization of DEI (n = 37)*	“Institutional value of community partnered research” “Set an agenda to make it a priority” “More discussion with PIs about expanding this mission at our institute” “Devise metrics that count towards advancement for investigators” “Better measures of change in cultural environment”
*Awareness, Understanding, and Knowledge (n = 21)*	“Stop treating health disparities work as not scientific” “Access and inclusion on all platforms”
*DEI Faculty and Staff Outreach, Recruitment, Retention, and Advancement (n = 15)*	“Need to revise promotion criteria to reward DEI work.” “Recruitment of DEI interested faculty” “Fund more staff” “Development programming and coaching approach” “Meeting seminars to demonstrate models”
*Community Engagement or Lack of Trust in Community (n = 14)*	“Build ongoing stable relationships with community-based organizations and leaders” “Institutional value of community partnered research”
*Networking and Collaboration (n = 11)*	“Create platform where CTSAs can identify researchers and areas of specialty and interest in collaboration”
*Other (n = 25)*	“Special issue in Translational Science” “Focus on less descriptive and more implementation efforts at scale” “Two way, equitable conversation” “Talk about how this research is impacting people’s health in plain language – discuss a population’s strength”
***Workforce Development***	
**-- Barriers (*n* = 162)**	
*DEI Faculty and Staff Outreach, Recruitment, Retention, and Advancement (n = 61)*	“Minimal diversity at the institution” “Limited diversity of applicant pool” “Not having special outreach for inclusion of diverse applicants” “Many unarticulated rules – easy to become an outsider” “I can only offer suggestions; I am not in a position in which I can directly effect change”
*Institutional and CTSA Commitment, Support, and Prioritization of DEI (n = 26)*	“Commitment of institutional leadership” “Lack of focus/prioritization on DEI” “One and done approach to education/training programs”
*Limited Funding (n = 20)*	“Lack of funding for pipeline programs…” “Money to bring opportunities to under-resourced, URM communities”
*Awareness, Understanding, and Knowledge (n = 7)*	“Untrained leadership” “Lack of awareness among underrepresented minority communities”
*Other (n = 14)*	“Concerns that applicants may consider diversity questions as biased” “Team is overwhelmed with just collecting data, not even close to stage of considering how we use it or how to revise from a DEI lens” “Location”
**-- Priorities or Suggestions (*n* = 149)**	
*DEI Faculty and Staff Outreach, Recruitment, Retention, and Advancement (n = 58)*	“Outreach to leaders in diverse communities” “Develop diverse pipeline from early in education/training path” “Developing relationships with HBCUs, K-12 pipelines” “Create a pipeline for recruitment, i.e., institutional and CTSA internships, grant support DEI” “Creating career paths to promote retention and promotion” “Guarantee two diversity supplements to each hub annually” “Invest early, continuity of focus on retention” “Increase visibility of STEM career opportunities early in educational process (elementary school)” “Recruit and retain more mentors who reflect underrepresented communities” “Reconceptualization for a realistic trajectory for many URM and focus on talent development and not talent selection” “Building bridges with other diversity programs for students at all levels on campus”
*Awareness, Understanding, and Knowledge (n = 23)*	“Cultural competency training” “Peer training from POC”
*Institutional and CTSA Commitment, Support, and Prioritization of DEI (n = 20)*	“University HR policies and procedures need an entire overhaul from the top down” “Develop and celebrate metrics associated with bringing on early career trainees into NIH diversity supplements.” “Training awards that buy-out 40% effort” “Do more to promote and support careers of URM minority”
*Funding and Resources (n = 18)*	“Encourage career in translational research with better financial support, subsidized day care opportunities, subsidized housing, and loan forgiveness programs” “Focused/dedicated funding and incentives for URM recruitment and retention” “Increase funding resources with less restrictions” “Internships and career development” “Fairness in access to resources”
*Networking and Collaboration (n = 8)*	“Exchange of ideas across institutions” “Share training resources across CTSA hubs”
*Other (n = 20)*	“Improve equity – economic and educational == across the US” “Tools or guides”
***CTSA Consortium Leadership***	
**-- Barriers (*n* = 127)**	
*DEI Faculty and Staff Outreach, Recruitment, Retention, and Advancement (n = 66)*	“Limited pool [of diverse applicants to leadership]” “Lack of URM in top leadership positions” “The majority of PIs are not diverse” “Lack of diversity within the CTSA sites to promote to leadership” “As staff, I do not have the capacity to influence DEI policy, governance, resources, and decision-making” “My position” “Hierarchical structures and systems” “Implicit bias in according authority” “Overarching metrics focusing on scholar productivity provide a disincentive for taking on diverse candidates that might be viewed as higher risk or in need of longer development times”
*Institutional and CTSA Commitment, Support, and Prioritization of DEI* *(n = 59)*	“Lack of focus on solutions” “Verbal support, but little/no systematic change, even as COVID has underscored the need” “Need all cores to make [DEI] a priority” “Recognition that this is an issue” “Lack of transparency” “Limited by not having DEI as a stated measure with NCATS” “Time required to absorb the volume of valuable information”
*Funding and Resources (n = 6)*	“There is a need for more minority supplements” “Resources to minority research leaders”
*Other (n = 7)*	“National: Ever changing priorities and lack of true commitment to improving population/community health” “No pressure to truly collaborate on things that matter”
**-- Priorities or Suggestions (*n* = 114)**	
*DEI Faculty and Staff Outreach, Recruitment, Retention, and Advancement (n = 99)*	“Create opportunities for mid-career faculty to engage in leadership and grow to take on more responsibility” “Don't tap into all the faculty of color for leadership – then they do not time for their research” “Adding more minority leaders as CTSA directors” “Intentional diversification of faculty affiliated with CTSA” “Enabling the next generation of more diverse leaders” “Sponsor junior faculty from diverse backgrounds to grow as leaders” “Prioritize diversity in our leadership thru mentoring and training” “Faculty hiring and leadership development, taking chances” “Intentional mentoring and recruitment of URM in leadership positions” “Offer leadership development to women and URM, & nurture/mentor emerging leaders”
*Institutional and CTSA Commitment, Support, and Prioritization of DEI (n = 29)*	“Raising the voices of more diverse leaders and their allies” “Broaden the conversation to include program staff” “Communication more broadly to all members of CTSAs – not just leadership” “Working groups” “Change in leadership” “Efforts to directly influence university and academic health center leadership to prioritize diversity” “Require that every image of CTSA includes broad diverse images, creative inclusive environment at all events. Adding support for a diversity officer at each hub.”
*Networking and Collaborations (n = 22)*	“Virtual office hours, town hall meetings, site visits to target populations” “Provide access – there were core forums in the past with monthly calls that allowed peer administrators at the core level to engage in meaningful discussion & sharing best practices” “We really should have a group dedicated to DEI” “Coordinated efforts and multi-group collaborations”
*Funding and Resources (n = 4)*	“Significantly augment number of minority supplements”
*Other (n = 11)*	“Streamline processes so time spent is more valuable” “Allow SC participation of non-PIs, reduce the effort requirements to be lead PI”
***Clinical Trials Participation (n = 125)***	
**-- Barriers (*n* = 140)**	
*Community Engagement or Lack of Trust in Community (n = 42)*	“Lack of trust in research institutions among URMs” “Building trust with the community after hundreds of years of medical distrust” “Suspicion by community members” “Lack of community engagement at the outset” “Access to special populations” “Increasing access to diverse populations” “Limited diversity in populations seen at medical center”
*Awareness, Understanding, and Knowledge (n = 28)*	“Awareness / skills needed to engage community” “Cultural and values knowledge” “Efforts need to be led by individuals who best represent specific trial populations and to involve communities in the research at hand” “It is difficult to make the direct link/benefit to the community” “Exploring novel recruitment strategies, dealing with barriers in language” “No one has talked about why there are so few BIPOC populations participating in clinical trials”
*Limited Resources (n = 23)*	“Limited resources for recruitment” “Inadequate resources to navigate the complicated agreements, budget negotiations, etc. required to participate in clinical trials” “Time and resources to appropriate engage in dialogue with underserved populations” “Lack of resources to transform clinical trials to have a DEI lens” “Representation of "people who look like" the minorities we seek “Lack of diversity among research staff
*Institutional and CTSA Commitment, Support, and Prioritization of DEI (n = 19)*	“Lack of value provided to diverse participants and lack of ask to participate” “Insufficient mandate to PIs to comply with equitable trial recruitment” “Study coordinators frequently not on board with increasing diversity” “Multiple competing programs at the institution with limited coordination” “Institutional racism”
*Limited Funding (n = 10)*	“Many of the trials are performed independently of the CTSA at our institution, since NCATS limits using our grant funds to support clinical research and clinical trials operations” “Reimbursement” “Lack of funding for recruitment specialists and support personnel to engage with URM communities”
*Other (n = 21)*	“Limited NIH and AHC institutional investment on the recruitment and retention of vulnerable/underserved/marginalized population groups” “Trials are run through the hospitals; university-based CTSC members have little influence over these practices and protocols”
**-- Priorities or Suggestions (*n* = 125)**:	
*Identify Innovative Recruitment Strategies (n = 24)*	“Access to information for participation on all platforms” “Better recruitment strategies – registries do not work” “Using innovative modern tools for clinical trials recruitment” “Enhance recruitment efforts through novel methods to ensure adequate representation of URMs and diverse gender and sexual orientations” “Central databases within and among our CTSA partners”
*Collaboration with Partners in Community (n = 18)*	“Get diverse community leaders involved” “Mandate full engagement of diverse community stakeholders in design and implementation” “Engaging community leaders” “Be more inclusive of community partners, ask them how to better include them in this process”
*Health Education Outreach (n = 18)*	“Saturate media and social medial with accurate information about the benefits of participating” “Implement more community outreach and training” “Mini research bootcamps to educate communities regarding actual research process”
*Building Relationships and Improving Community Perceptions (n = 17)*	“Building trust” “Concerted effort to restore trust and transparency within URM communities” “Seeking input from the community on ways to build trust and implementing them” “Meaningful investment in community wellness to build trust with research institutions” “Build ongoing stable relationships with community-based organizations and leaders”
*Institutional and CTSA Commitment, Support, and Prioritization of DEI (n = 15)*	“Need PIs to understand how to diversity their recruitment” “Institutional infrastructure and commitment to true community engagement” “Increase recognition and support for the engagement and bidirectional long-term relationships that are needed to build trust with underserved communities” “Set affirmative goals for diversity of participation for every trial” “Reward study coordinators who shine at inclusive enrollment, and talk with them to learn what they are doing” “Identifying benchmarks for diverse trials participation”
*More Funding (n = 13)*	“Reasonable reimbursement for studies” “Need more funding of minority PIs” “NCATS should allow UL1 budget to be used to offset costs of clinical research and investigator initiated clinical research and trials at our institutions” “Assets for sustained community partnerships”
*DEI Faculty and Staff Outreach, Recruitment, Retention, and Advancement (n = 9)*	“Create a workforce that looks like the participants we need” “Recruitment of minority faculty with this interest” “Track diversity among research staff”
*Networking and Collaboration (n = 7)*	“We are in a very diversified area of [CITY]. We can be approached to help others with our strategies” “Talk about how to get more BIPOC populations involved in clinical trials” “Use what has already been done to address barriers”
*Altering Study Design (n = 6)*	“More patient-centered clinical trial design” “Design clinical trials that allow disadvantaged people to participate”
*Awareness, Understanding, and Knowledge (n = 4)*	“Mandatory training for PIs and research coordinators in techniques for increasing diversity representation in clinical trials”
*Other (n = 9)*	“Break down racial barriers”

DEI = Diversity, Equity, and Inclusion; CTSA = Clinical and Translational Science Award; NCATS = National Center for Advancing Translational Sciences; NIMHD = National Institute on Minority Health and Health Disparities; HBCUs = Historically Black Colleges and Universities; STEM = Science, Technology, Engineering, and Mathematics; URM = Underrepresented Minorities; POC = People of Color; HR – Human Resources; NIH = National Institutes of Health; AHC = Academic Health Center.

*Note.* Comments appear as submitted. Identifying information is redacted in [brackets].

***Disparities and Health Equity Research*.** Regarding the barriers to Disparities and Health Equity Research, a total of 138 responses were received and 5 themes emerged. The top three themes were *Funding and Resources* (*n* = 42; e.g., “Limited money to support the high level of interest and competence among faculty and community partners to conduct partnered research.”), *Institutional and CTSA Commitment, Support, and Prioritization for DEI* (*n* = 26; e.g., “Lack of time/priority from top down – usually existing faculty working extra to meet a need”), and *DEI Faculty and Staff Outreach, Recruitment, Retention, and Advancement* (*n* = 24; e.g., “Lack of researchers engaged in this field” and “current lack of diversity in faculty”). On the priorities or suggestions for Disparities and Health Equity Research, a total of 135 responses were received and 6 themes emerged. The top three themes were *Funding and Resources* (*n* = 41; e.g., “Explicit funding mechanism outside of NIMHD”), *Institutional and CTSA Commitment, Support, and Prioritization for DEI* (*n* = 37; e.g., “institutional value of community partnered research” and “set an agenda to make it a priority”), and *Awareness, Understanding, and Knowledge* (*n* = 21; e.g., “Stop treating health disparities work as not scientific”).

***Workforce development*.** For the barriers to Workforce Development, a total of 162 respondents submitted comments. The three themes were *DEI Faculty and Staff Outreach, Recruitment, Retention, and Advancement* (*n* = 61; e.g., “limited diversity in applicant pool” and “many unarticulated rules – easy to become outsider”), *Institutional and CTSA Commitment, Support, and Prioritization for DEI* (*n* = 26; e.g., “one and done approach to education/training programs”), and *Limited Funding* (*n* = 20; e.g., “Lack of funding for pipeline programs…” and “money to bring opportunities to under-resourced, URM communities”). For priorities or suggestions, there were a total of 149 comments and 5 themes emerged. The top three themes were *DEI Faculty and Staff Outreach, Recruitment, Retention, and Advancement (n* = 58; e.g., “outreach to leaders in diversity communities” and “develop diverse pipeline…early in education/training path” and “developing relationships with HBCUs, K-12 pipelines”), *Awareness, Understanding, and Knowledge* (*n* = 23; e.g., “cultural competency training” and “peer training from POC”), and *Institutional and CTSA Commitment, Support, and Prioritization for DEI* (*n* = 20; e.g., “develop and celebrate metrics associated with bringing on early career trainees into NIH diversity supplements” and “training awards that buy-out 40% effort”).

***CTSA consortium leadership*.** For the domain of CTSA consortium leadership, a total of 127 respondents submitted comments on barriers, which yielded 3 themes. These included *DEI Faculty and Staff Outreach, Recruitment, Retention, and Advancement (n* = 66; e.g., “lack of diversity within the CTSA sites to promote to leadership” and “the majority of PIs are not diverse”), *Institutional and CTSA Commitment, Support, and Prioritization of DEI* (*n* = 59; e.g., “verbal support, but little/no systematic change” and “need all cores to make [DEI] a priority”), and *Funding and Resources* (*n* = 6; e.g., “there is a need for more minority supplements”). For the priorities or suggestions, 114 respondents submitted comments and 4 themes emerged. The top three themes included *DEI Faculty and Staff Outreach, Recruitment, Retention, and Advancement* (*n* = 99; e.g., “prioritize diversity in our leadership thru mentoring and training”), *Institutional and CTSA Commitment, Support, and Prioritization of DEI* (*n* = 29; e.g., “raising the voices of more diverse leaders and their allies”), and *Networking and Collaborations* (*n* = 22; “there were core forums in the past with monthly calls that allowed peer administrators at the core level to engage in meaningful discussion and sharing best practices”).

***Clinical trials participation*.** For the barriers to clinical trials participation, 140 respondents submitted comments and 5 themes emerged. The top three themes were *Community Engagement or Lack of Trust in Community* (*n* = 42; e.g., “suspicion by community members” and “lack of community engagement at the outset”), *Awareness, Understanding, and Knowledge* (*n* = 28; e.g., “cultural and values knowledge” and “efforts need to be led by individuals who best represent specific trial populations and to involve communities in the research at hand”), and *Limited Resources* (*n* = 29; e.g., “inadequate resources to navigate the complicated agreements, budget negotiations, etc., required to participate in clinical trials”). For the priorities or suggestions, 125 respondents submitted comments and 10 themes emerged. The top three themes were *Identify Innovative Recruitment Strategies* (*n* = 24; e.g., “enhance recruitment efforts through novel methods to ensure adequate representation of URMs and diverse gender and sexual orientations”), *Collaboration with Partners in Community* (*n* = 18; e.g., “mandate full engagement of diverse community stakeholders in design and implementation”), and *Health Education Outreach* (*n* = 18; e.g., “mini research bootcamps to educate communities regarding medical research process”).

## Discussion

The NIH [2] has an overarching interest in DEI as critical means for “fostering scientific innovation, enhancing global competitiveness, contributing to robust learning environments, improving the quality of the research, advancing the likelihood that underserved or health disparity populations participate in, and benefit from health research, and enhancing public trust.” This study sheds light on the attitudes towards DEI among leaders of the CTSA programs, a particularly important group for advancing DEI as CTSAs are designed to produce “innovative solutions that will improve the efficiency, quality and impact of the process for turning observations in the laboratory, clinic and community into interventions that improve the health of individuals and the public” [1].

Overall, the survey results can be viewed as reassuring, with nearly three out of four respondents (72.7%) reporting that DEI was “extremely important” to them, and an additional 20.8 percent reporting DEI as “very important.” This reflects how well DEI is incorporated into the stated values of the CTSA leadership. A deeper analysis, though, shows some concerning patterns. DEI was seen as “extremely important” by all NCATS officers (100.0%), with support progressively decreasing among TL1 Directors (86.7%), CTSA Program Hub Steering Committee Members (83.3%), KL2 Directors (77.3%), and UL1 Executive Directors/Administrators (71.4%) and was lowest among UL1 PIs (66.7%). Notably, the UL1 PIs and Executive Directors or Administrators were the only members of the CTSA consortium leadership group to have individuals reporting DEI only as “important” or “somewhat important.”

A similar pattern was seen in reported commitment to improving DEI through making fundamental changes in the way the consortium operates. The level of support was not as high overall, with 56.3 percent of all respondents reporting being “extremely committed,” and an additional 29.9 percent reporting “very committed.” Levels of commitment to change were highest among NCATS officers (71.4%), CTSA Program Hub Steering Committee Members (72.2%), and KL2 Directors (72.7%) and lowest levels among TL1 Directors (60.0%), UL1 Executive Directors/Administrators (57.1%), and UL1 PIs (56.7%). Once again, only the UL1 PIs and Executive Directors/Administrators had members reporting levels of “committed” or “somewhat committed.” These findings illustrate the need to elucidate how those in these positions can be moved towards committing to improving DEI. Furthermore, these individuals may need to be the focus of targeted efforts to understand and respond to their concerns and perspectives. As found in our study, respondents’ attitudes toward DEI matters, as perceived importance of DEI was positively associated with commitment to improving DEI through making fundamental changes. Accordingly, closing the gap between individuals’ perceived importance of DEI and their commitment to making improvements must accompany ongoing efforts to bolster DEI in the clinical and translational sciences and academic medicine.

To put these findings in context, leaders of academic medicine are increasingly expected to also be leaders in DEI and in the pursuit of health equity. The Association of Academic Medical Colleges (AAMC) has a goal of positioning itself as a national leader in health equity and health justice and has established a Center for Health Justice to support this effort. It has also developed principles, frameworks, and training programs to support this work, from which schools of medicine and health systems can utilize (see: https://www.aamc.org/addressing-and-eliminating-racism-aamc-and-beyond). Parallel efforts and programs are available from the National Academies of Sciences, Engineering and Medicine (NASEM; see: https://webassets.nationalacademies.org/healthequity/.). The CTSAs are part of this larger change across academic medicine, and it is particularly important that the leaders of the CTSAs not just support the importance of DEI but also be committed to making the changes needed. This includes actions such as working to ensure that the composition of institutional leaders, faculty, trainees, and staff reflect the diversity of the communities they serve, and that leaders are equipped with the perspective to recognize the value of equity work, such as mentorship to trainees [14]. The NIH has also promoted DEI as key to improving the quality and conduct of clinical and translational science [2]. Other key stakeholders, including non-NIH research professional organizations (e.g., Association of Clinical and Translational Research, the Clinical Research Forum, etc.) as well as the Editors-in-Chief of scientific journals (e.g., *Journal of Clinical and Translational Sciences*, *NEJM*, *JAMA*, *Preventing Chronic Disease*, etc.) have committed efforts to advance DEI efforts in science. Part of the reason it is so important for leaders in academic medicine to be committed to fundamental changes is because there are multiple barriers that must be overcome to improve DEI. In our study, the top barriers included: *DEI Faculty and Staff Outreach, Recruitment, Retention, and Advancement*; *Institutional and CTSA Commitment, Support, and Prioritization of DEI*; and *Funding and Resources*. As emphasized previously by Boulware and colleagues [14,15], combating structural inequities in clinical and translational research and realizing the vision of a truly diverse workforce called by the NIH will require bold steps. It is imperative for clinical and translational science organizations to transform individual perceptions of DEI into commitment, and – more importantly – commitment into action.

*DEI Faculty and Staff Outreach, Recruitment, Retention, and Advancement* touches upon the need to recruit more researchers from diverse backgrounds into clinical and translational sciences research. Furthermore, institutions must cultivate their workforce to match the communities they serve [14]. Efforts must include funding that adequately supports establishing programs and pathways (from high school to college and graduate programs) for recruiting and promoting researchers from underrepresented backgrounds. These endeavors must result in a more diverse investigator workforce, particularly at CTSA Program hubs [15]. Moreover, leadership must provide reassurance and demonstrate to their faculty and staff from underrepresented backgrounds that both their expertise and perspectives will be recognized and valued [15].

The *Institutional and CTSA Commitment, Support, and Prioritization of DEI* as well as the *Funding and Resources* themes emphasize the need for greater leadership among clinical and translational science organizations to promote and support DEI, either through establishing agendas or benchmarks for fostering DEI as well as putting resources behind such crucial efforts. For instance, there needs to be deliberate strategies developed geared toward recruiting and retaining participants from underrepresented groups for clinical research [15]. *Limited Funding and Resources* consistently appeared as a top theme in the current study, highlighting the need for additional investment in DEI. The *Workforce Development* area of interest generated the greatest amount of input from respondents, both with respect to the barriers and suggestions or priorities.

Leadership matters and the high levels of support for DEI reported here suggest that the CTSA Hubs can play important roles in helping transform research culture to support DEI within the hubs and their home institutions. Where that support and commitment lags, opportunities exist within the CTSAs themselves – and within academic medicine more broadly – to ensure that individuals have the opportunity to continue their growth and development so they can be the leaders that our students, faculty, institutions, and communities need and increasingly expect.

### Limitations

There are several limitations to this study. First, the response rate was 29 percent and attitudes of non-respondents were unknown. In addition, data were cross-sectional, which limited our ability to make causal inferences regarding the link between perceived importance of DEI and commitment to improving DEI given the absence of temporal associations. This self-reported survey may be affected by social desirability bias, as evidenced by the high number of respondents who indicated that DEI was at least very important to them or the proportion of those who indicated that they were at least very committed to improving DEI. Furthermore, the attitudes concerning DEI may not have been fully captured in this survey, as only two questions addressed this topic. Future research that more broadly assesses attitudes toward DEI with additional scaling options may better inform efforts to promote DEI in research institutions. However, limitations notwithstanding, we were the first to assess the perceptions of individuals currently active in CTSA hubs across the country. We also were able to ascertain specific actionable suggestions or priorities on how DEI can be improved across a broad range of domains of interest, including health disparities research, workforce development, CTSA consortium leadership, and clinical trials participation.

## Conclusion

As the COVID-19 pandemic has continued to exacerbate inequities across the USA, there is an urgent need for research geared towards addressing those structural inequities that continue to undermine progress in the clinical and translational sciences. Indeed, a collective shift in mindset is needed [16], compelling us to system-wide action and change that better aligns with the needs and the diversity of the communities that our institutions are charged to serve. Combating structural inequities in clinical and translational research, and realizing the vision of a diverse research workforce will require committed leaders and bold steps. Leaders in the clinical and translational sciences have begun to focus on DEI as an overarching priority, and this study showed that there is both a high level of agreement that DEI is important and a strong commitment to action, particularly among CTSA Program members. CTSA consortium leadership now needs to move forward in addressing structural inequities in clinical and translational research and including DEI as a core attribute of the CTSA Program.
